# Prognostic value of point-of-care ultrasound during cardiac arrest: a systematic review

**DOI:** 10.1186/s12947-020-0185-8

**Published:** 2020-01-13

**Authors:** Ilan Kedan, William Ciozda, Joseph A. Palatinus, Helen N. Palatinus, Asher Kimchi

**Affiliations:** 1 Smidt Heart Institute, Cedars-Sinai Medical Center, 8501 Wilshire Blvd. Suite 200, Beverly Hills, Los Angeles, CA 90211 USA; 2 Department of Emergency Medicine, McGovern Medical School, University of Texas Health Science Center at Houston, Houston, TX USA; 3 Department of Emergency Medicine, Cedars-Sinai Medical Center, Los Angeles, CA USA

**Keywords:** Ultrasound, Cardiac arrest, Point-of-care ultrasound, Echocardiogram, POCUS, Return of spontaneous, Circulation

## Abstract

**Background:**

Despite significant improvements in cardiopulmonary resuscitation, sudden cardiac arrest is one of the leading causes of mortality in the United States. Ultrasound is a widely available tool that can be used to evaluate the presence of cardiac wall motion during cardiac arrest. Several clinical studies have evaluated the use of ultrasound to visualize cardiac motion as a predictor of mortality in cardiac arrest patients. However, there are limited data summarizing the prognostic value of point of care ultrasound evaluation during resuscitation. We performed a systematic literature review of the existing evidence examining the clinical utility of point-of-care ultrasound evaluation of cardiac wall motion as a predictor of cardiac resuscitation outcomes.

**Methods/results:**

We performed a systematic PubMed search of clinical studies up to July 23, 2019 evaluating point-of-care sonographic cardiac motion as a predictor of mortality following cardiac resuscitation. We included studies written in English that reviewed short-term outcomes and included adult populations. Fifteen clinical studies met inclusion criteria for assessing cardiac wall motion with point-of-care ultrasound and outcomes following cardiac resuscitation. Fourteen of the fifteen studies showed a statistically significant correlation between the presence of cardiac motion on ultrasound and short-term survival. This was most evident in patients with ventricular fibrillation or ventricular tachycardia as a presenting rhythm. Absence of cardiac motion non-survival. The data were pooled and the overall pooled odds ratio for return of spontaneous circulation in the presence of cardiac motion during CPR was 12.4 +/1 2.7 (*p* <  0.001).

**Conclusion:**

Evaluation of cardiac motion on transthoracic echocardiogram is a valuable tool in the prediction of short-term cardiac resuscitation outcomes. Given the safety and availability of ultrasound in the emergency department, it is reasonable to apply point-of-care ultrasound to cardiopulmonary resuscitation as long as its use does not interrupt resuscitation.

## Background

Sudden cardiac arrest is one of the leading causes of mortality and morbidity in the United States despite significant cardiopulmonary resuscitation (CPR) efforts in the Emergency Department. Out of hospital cardiac arrest (OHCA) survival rates have improved with the recent increase in bystander CPR and automated external defibrillator (AED) application [1]. However, survival rates remain low. Nakahara et al. found that bystander witnessed CPR in OHCA increased one-month neurologic survival from 3.3 to 8.2% [1]. Similarly, the CARES registry, following OHCA in the US, found that survival to hospital discharge improved from 5.7% in 2005 to 8.3% in 2012 [2]. While the survival rate has improved over recent years, opportunities for meaningful progress in outcomes still remain.

**Fig. 1:**
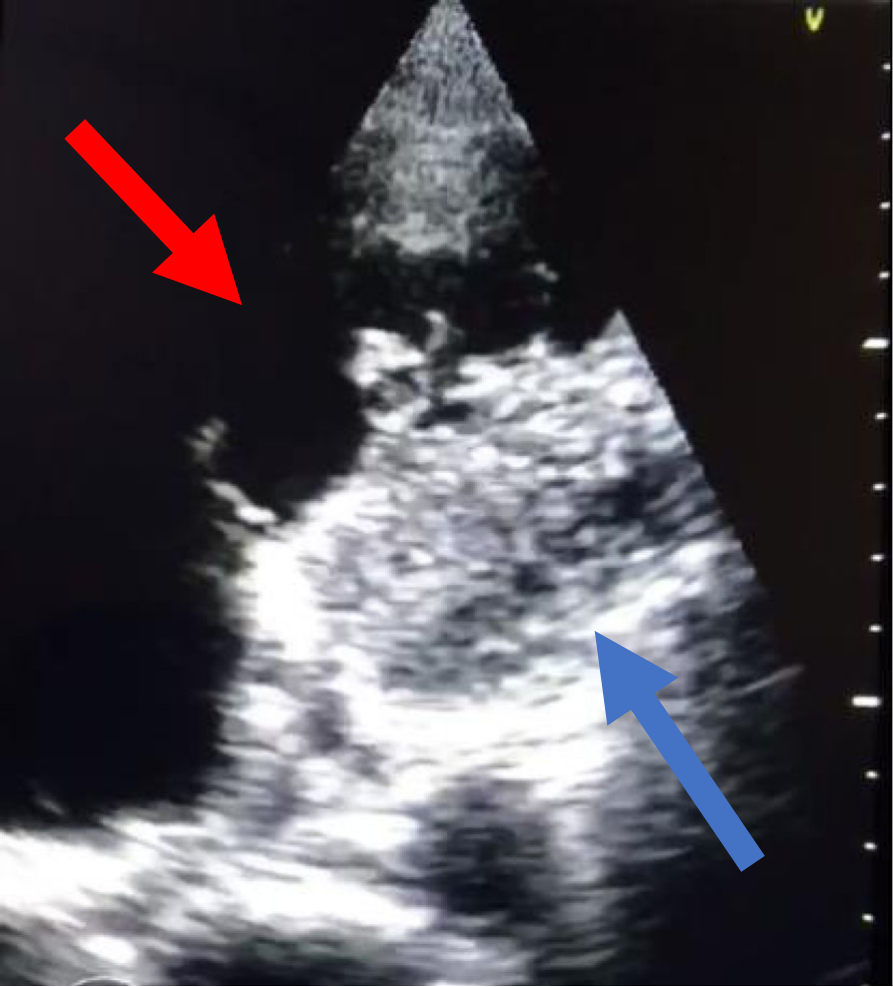
Massive pulmonary embolism in cardiac arrest

**Fig. 2:**
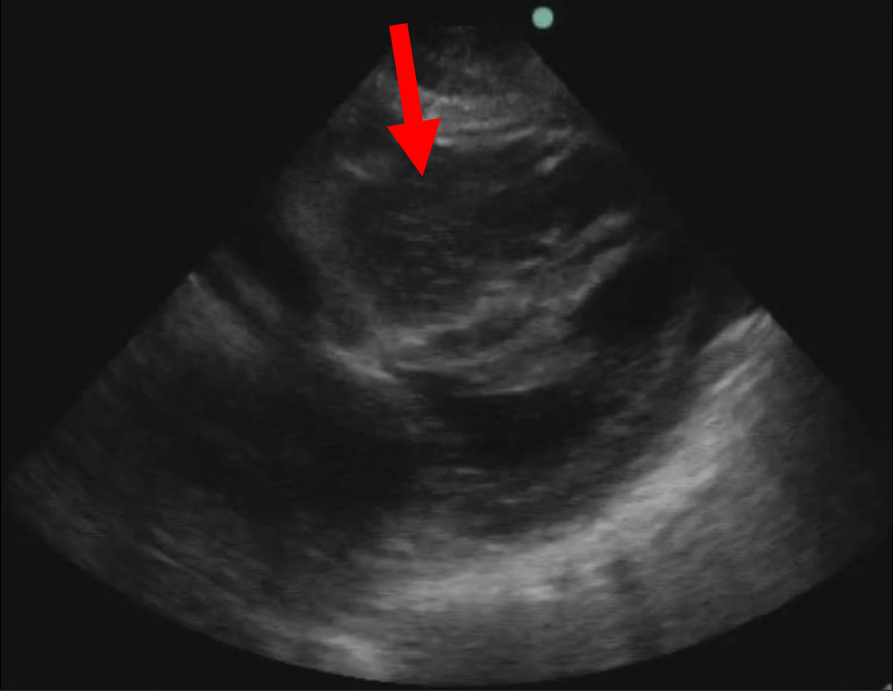
Coagulum formation in right ventricle with underfilled left ventricle in cardiac arrest

As point-of-care ultrasound (POCUS) has gained popularity by physicians in the emergency department (ED) and other clinical settings. POCUS has been shown to demonstrate real time physiologic data reflecting dynamic changes in response to medical treatments. Additionally, it has been shown to offer prognostic information in patients with heart failure and atrial fibrillation. Additionally, this technology has begun to be used to aid in the assessment of patients with cardiac arrest. Ultrasound is used by physicians for both the etiologic diagnosis in cardiac arrest patients as well as in the prognosis of resuscitation outcomes. POCUS has been shown as favorable in assessing for the presence of reversible causes of cardiac arrest including cardiac tamponade, hypovolemia, myocardial infarction and pulmonary embolism [3]. The identification of reversible etiologies by ultrasound has been shown to change subsequent management in the acute setting and will ideally be included in evidence-based resuscitation protocols in the future (Fig. 1) (Fig. 2). By better understanding the pathophysiology and natural history of cardiac arrest, POCUS may allow for better insights into the prognosis of patients receiving CRP following OHCA.

Several clinical studies have evaluated the use of cardiac motion visualized with point-of-care ultrasound as a predictor of outcomes in cardiac arrest patients. We provide a systematic review of the existing literature to examine the reliability and accuracy of cardiac motion visualized with point-of-care ultrasonography as a predictor for mortality outcomes in cardiac arrest patients.

## Methods

We performed a systematic literature search in PubMed with search terms of “Emergency Ultrasound in Cardiac Arrest” And “Emergency Sonography in Cardiac Arrest” to identify primary clinical research studies that compared mortality outcomes in cardiac arrest resuscitations with and without the use of POCUS. Only clinical studies with adult populations that pertained to our objective and were published in English prior to June 23, 2019 were included in our review. For each study, we collected data on study design, patient population as well as major findings. Excluded from review were studies with outcomes unrelated to cardiac arrest, pediatric and fetal populations, and reviews, case reports and letters. Outcome data from all studies were pooled, and the Mantel-Haenszel test was utilized to estimate the odds of return of spontaneous circulation in the presence of detectable cardiac motion across all studies. Data were compiled and displayed using Excel (Microsoft corporation) and Graphpad Prism 8 (Graphpad Software inc).

## Results

The initial literature review returned 729 journal articles. Of these, six pediatric and fetal ultrasound studies were excluded. Ninety-eight were excluded for being either a review article, case report, guideline article, editorial or letter and not a primary research study. Five were non-English publications. Six hundred twenty were deemed unrelated to the review objective. The fifteen remaining studies assessed cardiac movement with POCUS and outcomes after cardiac arrest resuscitation. The sample sizes ranged from 28 to 793 with a total of 2471 patients among all studies.

**Fig. 3:**
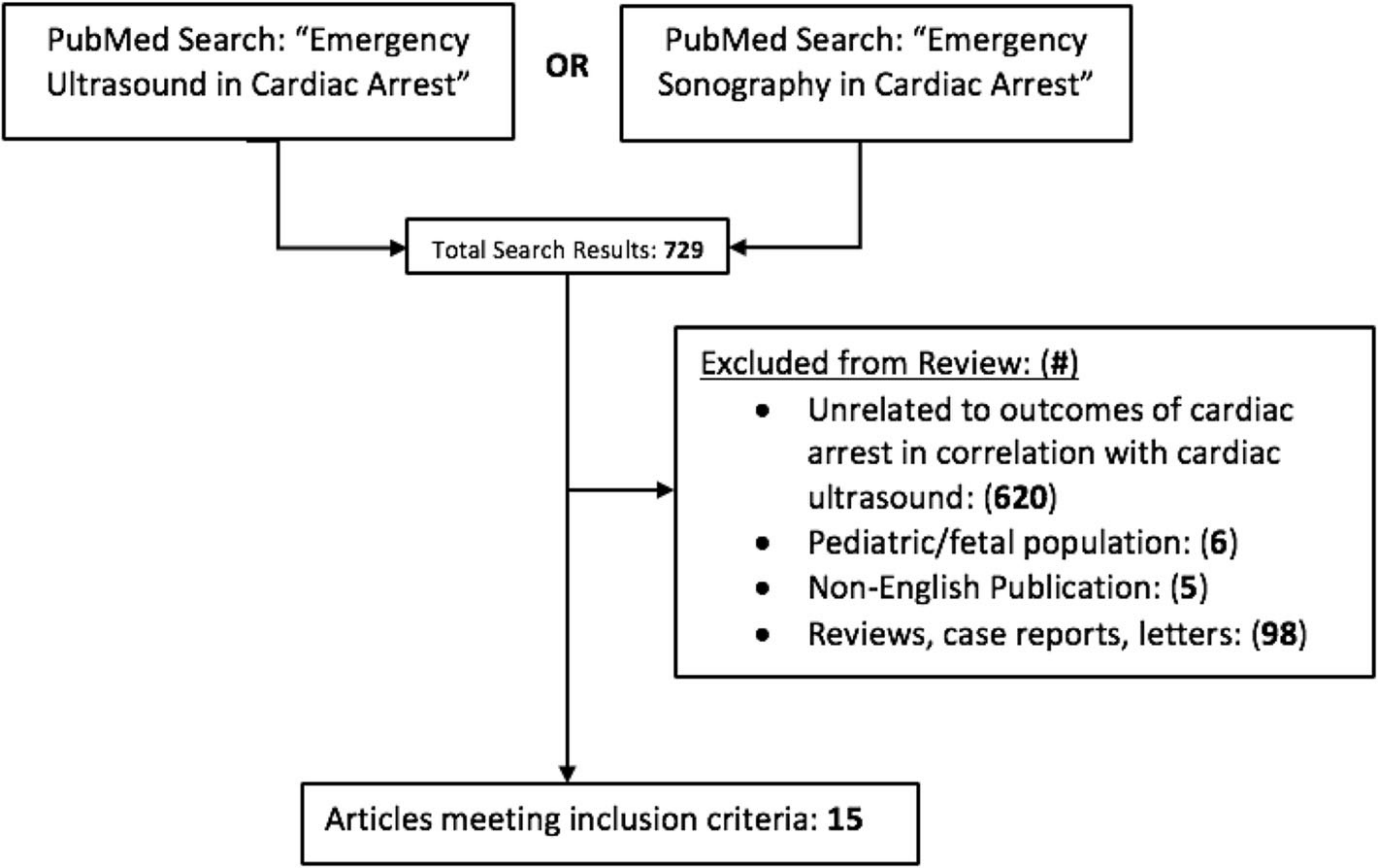
Study selection flowchart showing PubMed search and exclusion criteria

Selection criteria did not specify the use of trans-thoracic echocardiogram (TTE) or trans-esophageal echocardiogram (TEE). However, only studies utilizing TTE were found. All studies utilized the subxiphoid (subcostal) acoustic window for ultrasound image acquisition, either as the sole ultrasound window or in combination with additional windows. The additional acoustic windows included the parasternal, transthoracic and apical views. Imaging occurred in the ED in 13 studies and in the pre-hospital setting in the remaining 2 studies. Differences between studies include the probe type used, the types of cardiac arrest included, initial presenting cardiac rhythm and the timing of image acquisition during CPR. Imaging was performed upon ED arrival, upon initiation of resuscitation or during pauses in resuscitation. The definition of presence of cardiac motion was not standardized. (Fig. 3) Each study only loosely describes criteria for cardiac motion with a definition that differed between studies (Table 1).

**Table 1:** Summary of studies’ definition of cardiac motion

Study	Year	N	Definition of Cardiac Movement
Bolvardi et al.	2016	159	Not specified
Gaspari et al.	2016	793	any myocardial movement
Blaivas et al.	2001	173	No myocardial movement for 5–10 s
Salen	2001	102	Not specified
Ozen et al.	2016	129	Ventricular Wall Motion
Kim et al.	2016	48	any motion of atria, valvular, or ventricular
Cebicci et al.	2014	410	Not specified
Chardoli et al.	2012	50	Not specified
Tomruk et al.	2012	149	any motion of atria, valvular, or ventricular
Aichinger et al.	2012	42	any detected motion of myocardium
Breitkreutz et al.	2010	88	Not specified
Schuster et al.	2009	28	organized contractile activity (nonfibrillating) decrease in chamber size
Salen	2005	70	any motion of atria, valvular, or ventricular
Hayhurst et al.	2011	50	Not specified

Fourteen of the fifteen studies found a statistically significant positive correlation with sonographically visualized cardiac motion on point-of-care ultrasound and positive resuscitation outcomes (Table 2). The studies examined several different outcomes.

**Table 2:** Reviewed studies with association of cardiac motion on positive outcome. ROSC (return of spontaneous circulation)

Study	Outcome	Outcome *P*-Value
Bolvardi et al. [4]	ROSC	0.001
Gaspari et al. [5]	ROSC	< 0.001
–	Survival to Hospital Admission	< 0.001
–	Survival to Hospital Discharge	0.04
Salen et al. (2001) [6]	Survival to Hospital Admission	< 0.001
Ozen et al. [7]	ROSC	< 0.001
–	Survival to Hospital Admission	< 0.001
Cebicci et al. [8]	24 h survival	0.001
Chardoli et al. [9]	ROSC	N/S
Tomruk et al. [10]	ROSC	0.017
Aichinger et al. [11]	Survival to Hospital Admission	0.008
Salen et al. (2005) [12]	ROSC	0.05
–	Survival to Hospital Admission	< 0.05
Hayhurst et al. [1]	ROSC	N/S
Kim et al. [13]	ROSC	0.001
Blaivis et al. [14]	Survival to Hospital Admission	N/S
Schuster et al. [15]	ROSC	0.008
Brieithkruetz et al. [16]	Survival to Hospital Admission	N/S

**Fig. 4:**
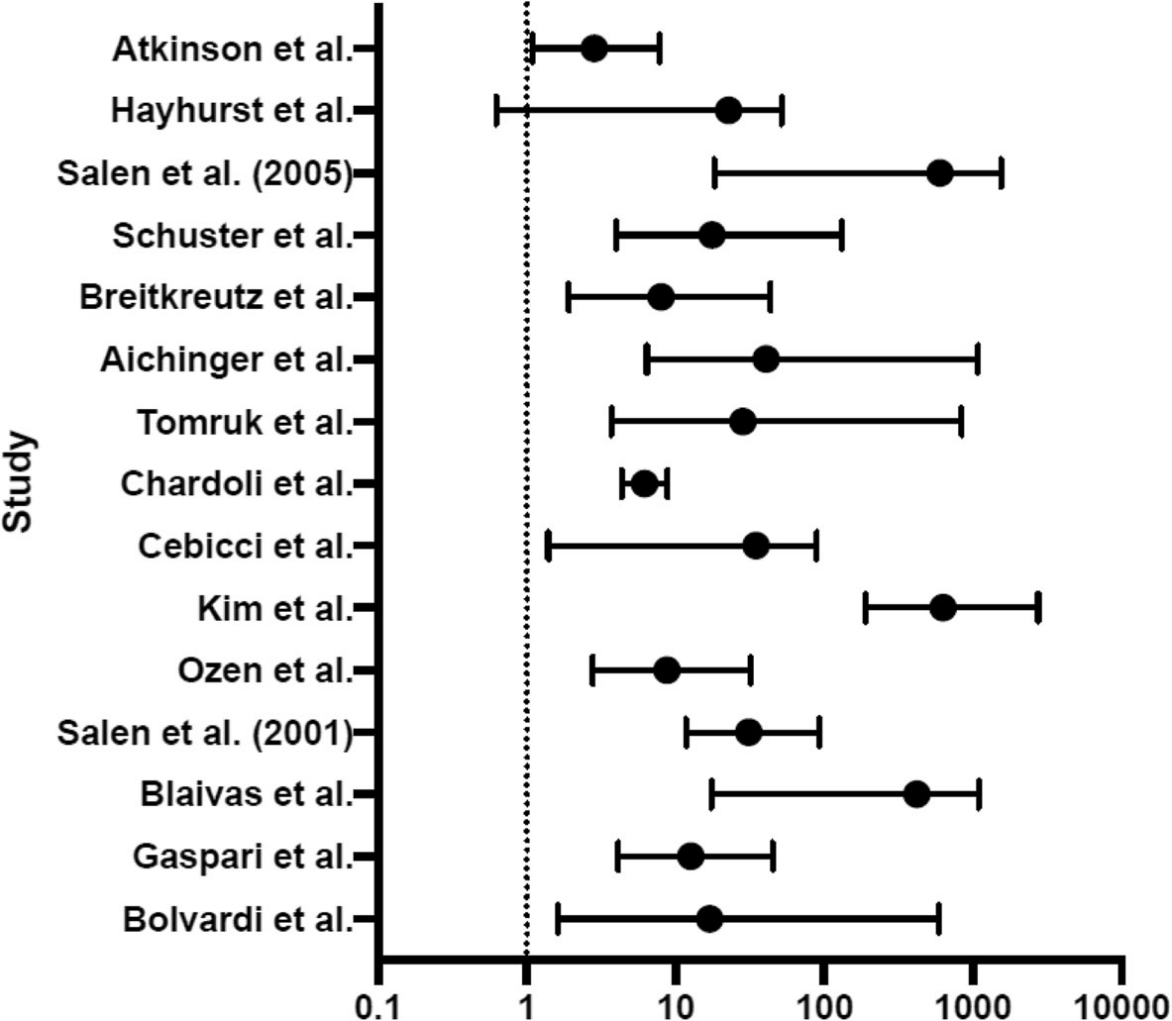
Forest Plot of odds of ROSC with detectd cardiac motion during cardiac arrest

A positive outcome was variably defined across studies as one of ROSC, survival to hospital admission, survival to hospital discharge or 24-h survival. Conversely, negative outcomes in patients noted to have cardiac standstill were also examined and summarized in Table 3. A negative outcome was defined as non-ROSC, death, non-survival to hospital admission or non-survival to hospital discharge. Of the 15 studies included in the review, all but one [6] demonstrated a statistically significant odds ratio between the presence of cardiac motion and ROSC. (Fig. 4).

**Table 3:** Percentage of patients that did or did not meet mortality outcomes stratified based upon whether cardiac motion was visualized on point-of-care ultrasound. Cumulative statistics also included

Study	Outcome	N	+ Outcome / + Cardiac Motion (*n*)	+ Outcome / - Cardiac Motion (*n*)	- Outcome / - Cardiac Motion (*n*)	- Outcome / + Cardiac Motion (*n*)
Bolvardi et al. [4]	ROSC	159	83.7% (41/49)	13.6% (15/110)	86.4% (95/110)	16.3% (8/49)
Gaspari et al. [5]	ROSC	793	51.0% (134/263)	14.3% (76/530)	85.6% (454/530)	49.0% (129/263)
–	Hospital Admission	793	28.9% (76/263)	7.2% (38/530)	92.8% (492/530)	77.1% (187/263)
–	Hospital Discharge	793	3.8% (10/263)	0.6% (3/530)	99.4% (527/530)	96.2% (253/263)
Blaivas et al. [14]	Hospital Admission	169	60.6% (20/33)	0% (0/136)	100% (136/136)	39.4% (13/33)
Salen et al. [6]	Hospital Admission	102	26% (11/41)	3.2% (2/61)	96.7% (59/61)	73.2% (30/41)
Ozen et al. [7]	ROSC	129	72.8% (56/77)	5.8% (3/52)	94.2% (49/52)	27.2% (21/77)
Kim et al. [13]	ROSC	48	87.5% (7/8)	52.5% (21/40)	47.8% (19/40)	12/5%(1/8)
Cebicci et al. [8]	24 h survival	410	91.4% (74/81)	15.2% (5/329)	98.5% (324/329)	8.6% (7/81)
Chardoli et al. [9]	ROSC	50	43% (17/39)	0% (0/11)	100% (11/11)	57% (22/39)
Tomruk et al. [10]	ROSC	149	70.4% (19/27)	45.1% (55/122)	54.9% (67/122)	29.6% (8/27)
Aichinger et al. [11]	Hospital Admission	42	40% (4/10)	3.1% (1/32)	96.9% (31/32)	60% (6/10)
Breitkreutz et al. [16]	Hospital Admission	88	58.8% (30/51)	13.5% (5/37)	86.5% (32/37)	41.2% (21/51)
Schuster et al. [15]	ROSC	28	25% (3/12)	0% (0/16)	100% (16/16)	75% (9/12)
Salen et al. [12]	ROSC	70	72% (8/11)	0% (0/59)	100% (59/59)	27% (3/11)
Hayhurst et al. [17]	ROSC	50	55% (11/20)	3% (1/30)	97% (29/30)	45% (9/20)
TOTAL		2291	60.2% (435/722)	11.8% (184/1565)	88.2% (1381/1565)	39.6% (287/722)

Pooled outcomes associated with the presence of cardiac motion studied were aggregated and are shown in Table 3. Outcomes are organized by the presence or absence of cardiac motion seen on POC ultrasound. 60.7% (451/743) of patients noted to have cardiac motion on point-of-care ultrasound met criteria for a positive outcome.

Conversely, 12.4% (215/1724) of patients without cardiac motion on point-of-care met criteria for a positive outcome.

These results were further organized by presenting cardiac rhythm. Pooled data from all studies were organized by the fraction of positive or negative outcome over the presence or absence of cardiac motion. These data were stratified according to the presenting rhythm of pulseless electrical activity (Table 4), Asystole (Table 5) and ventricular fibrillation or pulseless ventricular tachycardia (Table 6). Studies without presenting rhythm data were excluded from this stratification.

**Table 4:** Percentage of patients that presented with ONLY pulseless electrical activity as presenting rhythm that did or did not meet mortality outcomes stratified upon whether cardiac motion was visualized on point-of-care ultrasound. Cumulative statistics also included

Study	Outcome	N	+ Outcome / + Cardiac Motion	+ Outcome / - Cardiac Motion	- Outcome / - Cardiac Motion	- Outcome / + Cardiac Motion
Kim et al. [13]	ROSC	8	85% (6/7)	100% (1/1)	0% (0/1)	14.3% (1/7)
Chardoli et al. [9]	ROSC	50	43% (17/39)	0% (0/11)	100% (11/11)	57% (22/39)
Tomruk et al. [10]	ROSC	64	68.2% (15/22)	47.6% (20/42)	52.4% (22/42)	31.8% (7/22)
Schuster et al. [15]	ROSC	28	25% (3/12)	0% (0/16)	100% (16/16)	75% (9/12)
Salen et al. [12]	ROSC	34	72% (8/11)	0% (0/23)	100% (23/23)	27% (3/11)
Blaivas et al. [14]	Hospital Admission	38	67% (12/18)	0% (0/20)	100% (20/20)	33% (6/18)
Salen et al. [6]	Hospital Admission	55	25.8% (8/31)	4.1% (1/24)	95.8% (23/24)	74.% (23/31)
Aichinger et al. [11]	Hospital Admission	11	25% (1/4)	0% (0/7)	100% (7/7)	75% (3/4)
Breitkreutz et al. [16]	Hospital Admission	51	55% (21/38)	8% (1/13)	92% (12/13)	45% (17/38)
Cebicci et al. [8]	24 h survival	75	95.4% (42/44)	9.7% (3/31)	90.3% (28/31)	4.5% (2/44)
TOTAL		414	58.8% (133/226)	13.8% (26/188)	86.2% (162/188)	41.2% (93/226)

**Table 5:** Percentage of patients that presented with ONLY Asystole as presenting rhythm that did or did not meet mortality outcomes stratified upon whether cardiac motion was visualized on point-of-care ultrasound. Cumulative statistics also included

Study	Outcome	N	+ Outcome / + Cardiac Motion	+ Outcome / - Cardiac Motion	- Outcome / - Cardiac Motion	- Outcome / + Cardiac Motion
Salen et al. [12]	ROSC	36	0% (0/0)	0% (0/36)	100% (36/36)	0% (0/0)
Kim et al. [13]	ROSC	39	0% (0/0)	53.8% (21/39)	46.2% (18/39)	0% (0/0)
Tomruk et al. [10]	ROSC	77	80% (4/5)	43.1% (31/72)	56.9 (41/72)	20% (1/5)
Blaivas et al. [14]	Hospital Admission	65	0% (0/0)	0% (0/65)	100% (65/65)	0% (0/0)
Salen et al. [6]	Hospital Admission	36	0% (0/3)	3% (1/33)	97% (32/33)	100% (3/3)
Aichinger et al. [11]	Hospital Admission	20	100% (1/1)	5.3% (1/19)	94.7% (18/19)	0% (0/1)
Breitkreutz et al. [16]	Hospital Admission	37	69.2% (9/13)	16.7% (4/24)	83.3% (20/24)	30.8% (4/13)
Cebicci et al. [8]	24-h survival	290	0% (2/2)	0% (0/288)	100% (288/288)	0% (0/2)
TOTAL		600	66.7% (16/24)	10.1% (58/576)	89.9% (518/576)	33.3% (8/24)

**Table 6:** Percentage of patients that presented with ONLY ventricular fibrillation or pulseless ventricular tachycardia as presenting rhythm that did or did not meet positive mortality outcomes stratified upon whether cardiac motion was visualized on point-of-care ultrasound. Cumulative statistics also included

Study	Presenting Rhythm	Outcome	N	+ Outcome / + Cardiac Motion	+ Outcome / - Cardiac Motion	- Outcome / - Cardiac Motion	- Outcome / + Cardiac Motion
Kim et al. [13]	VT and VF	ROSC	1	100% (1/1)	0% (0/0)	0% (0/0)	0% (0/0)
Tomruk et al. [10]	VT and VF	ROSC	8	0% (0/0)	50% (4/8)	50% (4/8)	0% (0/0)
Blaivas et al. [14]	VF	Hospital Admission	66	53% (8/15)	0% (0/51)	100% (51/51)	47% (7/15)
Salen et al. [6]	VF	Hospital Admission	6	25% (1/4)	0% (0/2)	100% (2/2)	75% (3/4)
–	VT	Hospital Admission	5	67% (2/3)	0% (0/2)	100% (2/2)	33% (1/3)
Aichinger et al. [11]	VT and VF	Hospital Admission	9	66% (2/3)	0% (0/6)	100% (6/6)	33.3% (1/3)
Cebicci et al. [8]	VT and VF	24 h survival	45	85.7% (30/35)	20% (2/10)	80% (8/10)	14.3% (5/35)
TOTAL			140	72.1% (44/61)	7.6% (6/79)	92.4% (73/79)	27.9% (17/61)

A total of 414 patients, from eleven studies, were found to have pulseless electrical activity. Of these, 58.8% (133/226) of patients noted to have cardiac motion on POCUS and met criteria for a positive outcome. Conversely, 13.8% (26/188) of patients without cardiac motion on POCUS met criteria for a positive outcome of ROSC, survival to hospital admission or 24-h survival.

A total of 600 patients, from eight studies, had asystole as the presenting rhythm. (Table 5) Of these, 66.7% (16/24) of patients noted to have cardiac motion on POCUS and met criteria for a positive mortality outcome. Conversely, 10.1% (58/576) of patients without cardiac motion on POCUS met criteria for a positive outcome of ROSC, survival to hospital admission or 24-h survival. (Table 5).

A total of 140 patients, from six studies, were found to have with either ventricular fibrillation or ventricular tachycardia as the presenting rhythm. (Table 6) Of these, 72.1% (44/61) of patients noted to have cardiac motion on point-of-care ultrasound and met criteria for a positive mortality or morbidity outcome. Conversely, 7.6% (6/79) of patients without cardiac motion on point-of-care met criteria for a mortality or morbidity outcome of ROSC, survival to hospital admission or 24-h survival. (Table 6).

These data were further stratified according to outcome. Once again, data was organized by the percentage of positive or negative outcome over the presence or absence of cardiac motion. Only data for ROSC (Table 7) and survival to hospital admission (Table 8) was used as other the outcomes did not have sufficient power.

**Table 7:** Percentage of patients in which return of spontaneous circulation (ROSC) was or was not achieved during resucitation stratified upon whether cardiac motion was visualized on point-of-care ultrasound. Cumulative statistics also included

Study	Outcome	*N*	+ ROSC / + Cardiac Motion	+ ROSC / - Cardiac Motion	No ROSC / - Cardiac Motion	No ROSC / + Cardiac Motion
Bolvardi et al. [4]	ROSC/ROB/ROBP/ROP	159	83.7% (41/49)	13.6% (15/110)	86.4% (95/110)	16.3% (8/49)
Gaspari et al. [5]	ROSC	793	51.0% (134/263)	14.3% (76/530)	85.6% (454/530)	49.0% (129/263)
Ozen et al. [7]	ROSC	129	72.8% (56/77)	5.8% (3/52)	94.2% (49/52)	27.2% (21/77)
Kim et al. [13]	ROSC	48	87.5% (7/8)	52.5% (21/40)	47.8% (19/40)	12/5% (1/8)
Chardoli et al. [9]	ROSC	50	43% (17/39)	0% (0/11)	100% (11/11)	57% (22/39)
Tomruk et al. [10]	ROSC	149	70.4% (19/27)	45.1% (55/122)	54.9% (67/122)	29.6% (8/27)
Salen at al [6].	ROSC	70	72% (8/11)	0% (0/59)	100% (59/59)	27% (3/11)
Schuster et al. [15]	ROSC	28	25% (3/12)	0% (0/16)	100% (16/16)	75% (9/12)
Hayhurst et al. [17]	ROSC	50	55% (11/20)	3% (1/30)	97% (29/30)	45% (9/20)
TOTAL		1476	58.5% (296/506)	17.6% (171/970)	82.4% (799/970)	36.4% (210/576)

**Table 8:** Percentage of patients which did or did not survive to hospital admission stratified upon whether cardiac motion was visualized on point-of-care ultrasound. Cumulative statistics also included

Study	Outcome	*N*	+ Outcome / + Cardiac Motion	+ Outcome / - Cardiac Motion	- Outcome / - Cardiac Motion	- Outcome / + Cardiac Motion
Bolvardi et al. [4]	Hospital Admission	793	28.9% (76/263)	7.2% (38/530)	92.8% (492/530)	77.1% (187/263)
Gaspari et al. [5]	Hospital Admission	173	60.6% (20/33)	0% (0/136)	100% (136/136)	39.4% (13/33)
Salen et al. [6]	Hospital Admission	102	26% (11/41)	3.2% (2/61)	96.7% (59/61)	73.2% (30/41)
Aichinger et al. [11]	Hospital Admission	42	40% (4/10)	3.1% (1/32)	96.9% (31/32)	60% (6/10)
Breitkreutz et al. [16]	Hospital Admission	88	58.8% (30/51)	13.5% (5/37)	86.5% (32/37)	41.2% (21/51)
TOTAL		1198	35.4% (141/398)	5.7% (46/796)	94.2% (750/796)	64.6% (257/398)

Ten studies, with a total of 1656 patients, studied return of spontaneous circulation (ROSC) in relation to the presence of cardiac motion on POCUS (Table 7). The data from Bolvardi et al. was included which grouped ROSC with return or breathing, return of palpable pulse and return of measurable blood pressure. Of all patients assessed for ROSC, 59.2% (312/527) of patients noted to have cardiac motion on POCUS and met criteria for return of spontaneous circulation. Conversely, 17.9% (202/1129) of patients without cardiac motion on point-of-care did not meet criteria for ROSC. (Table 7).

Six studies, with a total of 1378 patients, studied survival to hospital admission in relation to the presence of cardiac motion on POCUS. (Table 8) Of all patients assessed for survival to hospital admission, 35.3% (148/419) of patients noted to have cardiac motion on POCUS survived to hospital admission. Conversely, 6.0% (46/955) of patients without cardiac motion on POCUS survived to hospital admission. (Table 8).

Using ROSC as the primary outcome, the results of all studies were pooled using the Mantel Haenzel test to calculate the odds ratio and 95% confidence intervals for the presence of cardiac motion and the odds of ROSC. (Fig. 4). The overall pooled odds ratio for ROSC in the presence of cardiac motion during CPR was 12.4 +/1 2.7 (*p* < 0.001).

## Discussion

Our review of the data demonstrates a consistent relationship between the presence of cardiac motion on POCUS during cardiac arrest and “positive” outcomes of cardiac resuscitation. As cardiac motion is required for any positive survival outcome, it is not surprising that the presence of cardiac motion is associated with survival during resuscitation efforts. With current guideline directed clinical practice recommendations, the presence of cardiac or absence of cardiac motion seen with ultrasound is not an indication to alter management decisions. The negative predictive value of cardiac motion on positive outcomes may impact clinical practice. Across all studies, the absence of cardiac motion on ultrasound results in non-ROSC in 82% of patients. Furthermore, 94% of patients with absent cardiac motion did not survive to hospital admission. This suggests the potential for clinical utility in assessing cardiac motion at the initiation of or during pauses in cardiac resuscitation as a screening measure for patients presenting to the ED in cardiac arrest.

Our review only included patients who received point-of-care echocardiograms either at the initiation of resuscitation or during the cardiac resuscitation. Jentzer et al. studied outcomes of cardiac arrest patients who underwent an echocardiogram within 24 h of hospital admission with an average time to ultrasound of 11.9 h [18]. The investigators found no relation between mortality and any echocardiographic markers of cardiac function including left ventricular ejection fraction and left ventricular relative wall thickness [18]. The lack of a statistical relationship with mortality between these more involved ultrasound variables assessed in this study underlines the potential value of POCUS in the acute clinical setting. The presence of cardiac motion is a binary and simple assessment. When performed at the point-of-care, ultrasound can contribute valuable clinical applications in predicting short-term outcomes.

Furthermore, the pooled data support the use of serial ultrasound imaging in the evaluation of cardiac activity. Kim et al. studied the outcomes compared to cardiac activity seen on initial ultrasound as well as serial ultrasounds performed at two-minute increments during pulse checks [13]. The positive predictive value of cardiac standstill on the first ultrasound was 46.5% to predict non-ROSC [13]. This number increased to 85.7% with cardiac standstill on 6 min of serial ultrasounds and 100% at 10 min of serial ultrasounds. As Kim et al. described, the presence of continuous cardiac standstill on ultrasound despite resuscitation efforts may be used as a marker to discontinue resuscitation efforts [13].

Most of the reviewed studies acquired echocardiographic data from the ED setting. However, two studies, Aichinger et al. and Briekreutz et al. examined echocardiographic cardiac motion in the pre-hospital setting using hand-held ultrasound devices [11, 16]. These two studies showed similar results to those studies performed in the ED. Aichinger et al. found cardiac standstill to have a positive predictive value (PPV) of 86% as a predictor of death [11] . Similar results in the pre-hospital setting show that adequate images can be obtained with more ultrasound devices in non-hospital settings. Furthermore, by obtaining images in the pre-hospital setting, pre-hospital teams may be able to provide the resuscitation team with additional clinical and prognostic information with the potential to modify clinical management decision-making.

POCUS has the potential for real-time assessment of physiologic changes that may result from medical therapies and interventions by observing dynamic anatomic findings that are seen with ongoing patient treatments [19, 20]. While the use of transthoracic echocardiogram has shown a great deal of utility and efficacy it does have some limitations. Most notably, TTE cannot easily or typically be used to evaluate cardiac activity during active cardiac resuscitation and is largely limited in use to prior to the initiation of resuscitation or during pauses in CPR. Trans-esophageal echocardiogram (TEE) may allow for continuous ultrasonographic visualization of cardiac activity throughout resuscitation efforts and could offer utility in informing the care team of clinical information while also predicting resuscitation outcomes [21] . To date, the use of TEE in the ED has been limited, there are data supporting the use of TEE to diagnose the potential etiology of cardiac arrest. Van Der Wouw et al. found that in 48 patients with cardiac arrest in the ED, TEE was able to make a definite diagnosis as to the etiology of the cardiac arrest with a sensitivity of 93%, specificity of 50% and PPV 50% [22]. TEE was able to diagnose cardiac tamponade, myocardial infarction, pulmonary embolism, aortic dissection, aortic rupture and papillary muscle rupture. In addition to aiding in diagnosis, TEE may be useful for the assessment of cardiac motion and prognosis during cardiac arrest. This may be more applicable to in-hospital cardiac arrest patients where TEE expertise and access may be more available than in current typical ED settings.

There are several limitations to the present study. Although we found a significant association between the presence of cardiac motion during resuscitation and ROSC, currently there is insufficient evidence to recommend abandoning resuscitative efforts based on the absence of cardiac motion alone. There are clearly certain instances when a lack of cardiac motion may not be predictive of failure of ROSC, especially in the setting of hypothermia or drug intoxication or in clinically induced cardiac standstill/cardiac arrest as in cardiac surgery for example [23]. Furthermore, it remains unclear from the studies reviewed if ROSC, survival to admission or even survival to hospital discharge is a meaningful clinical outcome. Patients who have received CPR and survive to hospital discharge may have significant neurological impairment and few studies characterized the role that POCUS may play in predicting favorable neurologic outcomes. Also, a survivor bias effect may be present for patients that were included in the reviewed studies as this cohort of patients may have possessed characteristics that favored more prolonged survival to inclusion in these research studies. Additionally, there may be relevant or similar studies that fell outside of the search criteria. The studies included do not specify or include the use of pocket-sized POCUS devices that are gaining popularity in clinical practice [24].

## Conclusions

With the increasing availability of affordable handheld POCUS devices, we believe there will likely be increased physician comfort and skill with the use of ultrasound in the ED and critical care setting. While these results suggest POCUS provides additional diagnostic and prognostic information in the management of cardiac arrest, with the current body of knowledge regarding POCUS in the setting of cardiac arrest, we believe it remains vitally important that point-of-care imaging does not interfere with standard advanced cardiac life support efforts. With further study and perhaps a randomized multicenter trial using POCUS during treatment of cardiac arrest, perhaps standardized POCUS data can be incorporated in the evidence-based treatment of patients suffering from cardiac arrest. Point-of-care assessment of cardiac motion has the potential to be informative as an additive clinical data point in the clinical assessment of patients suffering from cardiac arrest.

## Data Availability

Not Applicable.

## Abbreviations

AED: Automated external defibrillator
CPR: Cardiopulmonary Resuscitation
ED: Emergency Department
non-ROSC: No return of spontaneous circulation
OHCA: Out of Hospital Cardiac Arrest
PEA: pulseless electrical activity
POCUS: Point-of-care ultrasound
PPV: Positive predictive value
ROSC: Return of spontaneous circulation
TEE: Trans-esophageal echocardiogram
TTE: Trans-thoracic echocardiogram
VF: Ventriular Fibrillation
VT: Ventricular Tachycardia
